# Translation to Spanish and linguistic validation of the Canine Brief Pain Inventory

**DOI:** 10.3389/fvets.2023.1203453

**Published:** 2023-06-30

**Authors:** María Olcoz, Miguel Ángel Cabezas, Giorgia della Rocca, Ignacio A. Gómez de Segura

**Affiliations:** ^1^Department of Animal Medicine and Surgery, University Complutense of Madrid, Madrid, Spain; ^2^CeRiDA – Research Center on Animal Pain, Department of Veterinary Medicine, University of Perugia, Perugia, Italy

**Keywords:** chronic pain, pain assessment, osteoarthritis, dog, questionnaire

## Abstract

**Introduction:**

Pain scales for the assessment of chronic pain have been developed for dogs but they should be translated and linguistically validated to be used by owners with different native languages. The Canine Brief Pain Inventory (CBPI) is widely employed for this purpose but has not been translated into Spanish. Thus, the aim was to produce a validated translation of the Spanish CBPI.

**Methods:**

The original English version of the CBPI was analyzed and translated by two native linguists of the target language and both revised by a third native linguist to identify potential discrepancies and create a unified translation (reconciliation). Then, an independent linguist with native fluency in English and the target language drafted the back-translation. Finally, the research team confronted both the original and the back-translation to identify and solve relevant differences. Once the translated version was produced, a cognitive debriefing was performed to assess the questionnaire in the target population.

**Results:**

A total of 50 surveys were conducted to dog and cat owners of different ages, sex, and socio-economic characteristics. All respondents considered the survey to be clear and a final version of the Spanish CBPI has been produced.

## 1. Introduction

A relevant issue in the development and assessment of new pain treatments is the difficulty in a reliable pain assessment due to its complex, multidimensional and subjective nature (1). In addition, animals cannot verbalize the degree of pain, so its assessment must be done indirectly (2). In the case of chronic pain, considering that lasting for more than 3 months (3), the difficulty is greater: the associated clinical signs are more subtle, intermittent and, in most cases, with a slow onset that results in gradual changes in behavior (2). These characteristics limit the assessment of pain by the veterinarian since the patient can only be assessed during a specific time, and in a non-familiar setting (2).

Alternatively, there has been growing interest in the behaviourally based pain assessment provided by the owner, which allows continuous and long-term assessment in the animal’s usual environment. Disadvantages include increased subjectivity, where behaviors may be biased by the environment and the person assessing. For owner assessment of chronic pain in dogs and cats, clinical metrology instruments (CMI), also known in veterinary medicine as owner-reported outcome measures (OROM), have been developed (4). These CMIs are derived from human medical equivalents that collect information directly from patients (symptoms, health-related quality of life or functional status) (4, 5). They involve sequences of questions or items that are scored based on the observations or experiences of the person completing them, and the scores of these individual items are then used to calculate the overall score of the CMI (6). Among the CMIs described for assessing the impact of osteoarthritis in dogs is the Canine Brief Pain Inventory (CBPI) (6–8) or Chronic Pain Assessment Scale for Owners. This scale allows owners to rate the severity of their dog’s pain and the degree to which it interferes with function. This scale also allows assessment of pain caused by bone cancer (4, 7–9).

The CBPI has a clear language that allows for proper interpretation by owners even without technical veterinary knowledge. The problem arises when this questionnaire, originally in English, is passed on to owners whose native language is not English. The necessary translation from English to other languages presents difficulties, as it may not have the same meaning or may have different nuances, thus implying different assessments. Linguistic validation is the process by which the cultural appropriateness and conceptual equivalence of translated items is assessed and aims to ensure that the content validity of the original item has not been affected by translation (10). Translation and linguistic validation of the CBPI into different languages reduces the risk that the data obtained are invalid due to incorrect translation, and that differences in responses between populations are due to differences between groups rather than differences in how the data were collected (1).

While the CBPI has been translated and validated into Swedish (11), French (12), Italian (13), German, Dutch (Netherland), Hungarian, Chinese, Japanese (1), Portuguese (1, 14), English (Australia) and English (Ireland) (1), it has not been translated or validated into Spanish. Thus, the aim of this study was to develop a conceptually equivalent and culturally relevant version of the CBPI for use in Spanish. As a main hypothesis, it was established that a valid translation into Spanish spoken in Spain (from now on in the article Spanish will be considered as the language spoken in Spain) of the CPBI, culturally and conceptually equivalent to the original English version, can be obtained.

## 2. Materials and methods

Like the original CBPI version (7), the Spanish version should consist of a total of 10 items, four related to the severity of pain evident in a dog (severity domain) and six related to the interference of pain with daily activities (interference domain), which are measured using a numerical scale from 0 to 10. Finally, a global quality of life item is included to obtain the owner’s overall assessment of the dog’s quality of life status, measured by a categorical response from a 5-point scale.

For the translation into Spanish of the original English CPBI, the guidelines and recommendations established by the World Health Organization and the International Society for Pharmacoeconomics, and Outcomes Research (ISPOR) have been followed (10, 15, 16) (Appendix A). Also, the methodology used in the translation and validation of the CBPI into other languages (1, 11–14) was considered. Prior permission of the CBPI developer, Dr. Dorothy Cimino Brown, was also obtained.

Before translation, each element of the CBPI was analyzed in order to identify concepts, technical terms and phrases that could generate doubts or hinder the translation process. Besides, the need to draft a document for the translators with key terms, definitions, and a brief description of the CBPI was planned but considered unnecessary given the brevity and conciseness of the survey. The selected translators were verbally explained the recommendations for the translation, which consisted of a brief and concise translation, avoiding vulgarisms, colloquialisms or any other term that could cause confusion. Literal translations were to be avoided, as they could lose meaning or coherence in the target language. In this sense, priority was given to maintaining the original meaning of the text.

For the translation process, two native linguists of the target language, in this case Spaniards, were selected to carry out the direct translation (from English to Spanish), each independently. One of them was a veterinarian with technical knowledge and familiarity with the CBPI, while the other was a person with no relation to health sciences. Although the WHO and ISPOR recommend that direct translation should be carried out by healthcare professionals, a non-veterinarian was included to perform one of the direct translations. The two direct translations were then compared by a third native Spanish linguist (also Spaniard) to identify any discrepancies between both translations and to create a unified direct translation (reconciliation).

Then, an independent linguist with native fluency in English and the target language drafted the back-translation, that is, translated the unified Spanish document into the source language (US English).

Subsequently, the research team, as well as one of the linguists involved, conducted a thorough review to examine and resolve any discrepancies between the direct Spanish translation, the back-translation into English, and the original document, as well as to ensure the clarity of wording and translation concepts. When necessary, if relevant differences or any comprehension problems were identified in the target language, a new translation was carried out.

Once the translation process was completed, cognitive debriefing was performed to assess the questionnaire in the target population. Although the WHO and ISPOR recommend a minimum of 5 people to perform this cognitive debriefing, a total of 50 surveys were conducted with randomly selected dog and cat Spanish (Spain) speaking native owners of different ages, sex and socio-economic characteristics, in order to be representative of the target population. They were given a questionnaire with a brief description of the CPBI and the objective of this study and were asked to read the translated version of the CBPI, indicate whether each item or element was clearly understood, and suggest possible response alternatives (phrases or words) that they considered could facilitate the understanding of the question.

Finally, the research team evaluated all the questionnaires to assess whether any modifications were necessary to obtain a definitive translation of the CPBI into Spanish.

The Spanish version of the CBPI and the questionnaire used in the cognitive debriefing process are available as Supplementary material.

## 3. Results

The translation and linguistic validation process, as well as the cognitive analysis, allowed for the elaboration of a Spanish version of the CBPI conceptually equivalent to the original English version.

The direct or independent translations made by the two Spanish native linguists were very similar, although there were a total of 8 items or elements where there was some discrepancy between the two versions. Thus, the third native Spanish linguist compared and merged the two versions to produce the unified translation, selecting the terms in the translation that were considered most appropriate (considering the original version) or using different terms that were more similar to the original document. For example, the item “rate your dog’s pain” was translated as “*califica el dolor de tu perro*” and “*valore el dolor de su perro*,” however, the third linguist considered that “*puntúe el valor de su perro*” would be more appropriate. Another example is the item “…describes the pain at its worst in the last 7 days,” which was translated as “… *describa el dolor más extremo e intenso en los últimos 7 días*” and “… *describa el dolor en su peor momento en los últimos 7 días*.” The third linguist finally translated it as “… *describa el peor dolor en los últimos 7 días*,” thus avoiding the use of the terms “*intenso*” and “*momento*” which, although these terms would not imply a relevant change in the meaning and understanding of the question, do not appear in the original document.

In the back-translation, 11 items were identified as differing from the original version. The research team analyzed all discrepancies between the unified translation, the back-translation, and the original version and, whereas discrepancies remained, consulted with the native English-speaking linguist. Of these 11 items, only two were modified and retranslated. One example is the item “overall impression,” which was translated as “*impresión general* “and, in the back-translation, as “general impression.” The research team together with the native English linguist decided to use “*impresión global*,” since “general” and “overall” have a slightly different connotation, which could influence the responses. Once the translation process was completed, all discrepancies were resolved and the unified translation was finalized, a cognitive debriefing was carried out.

A total of 50 dog and cat owners were surveyed to assess the readability and comprehension of the Spanish version of the CPBI. Respondents were classified according to age range, sex, and level of education. Of these 50 respondents, 26 were male (52%) and 24 were female (48%). The age ranges included were: 20–29 years (18%), 30–39 years (26%), 40–49 years (18%), 50–59 years (18%) and ≥ 60 years (20%). The level of education of respondents was elementary and middle school (10%), high school (16%) and university graduate or higher (74%). In general, all participants understood each item of the CBPI, as well as the response options, without difficulty. However, there were 12 people (24%) who suggested changing a phrase or word to make it sound more natural.

Among the respondent’s suggestions, one person suggested changing the term “*justa*” to “*mediocre*” in the assessment of the dog’s quality of life, while another respondent suggested changing it to “*limitada*.” The authors finally decided to change the word “*justa*” to “*en el límite*.” This latter term (“*en el límite*”) better reflects the English concept (“fair”) since a more literal translation, “*justa*,” is misleading as it is more often used in terms of fairness, legal or even-handedness. The intended meaning is to be at the limit of what is acceptable, below which it would result in an unacceptable level of pain. The authors did not choose the terms “*mediocre*” and “*limitada*,” as both were considered to have a more negative connotation than the original English term. Another problematic item was “… the pain at its average,” translated as “… *el dolor medio*.” One respondent noted that he did not understand the concept of “*dolor medio*” and other suggested changing it to “… *el dolor normal*.” Upon review, the researchers decided to replace “*medio*,” more commonly used as a concept that implies a middle level between the owner’s assessments of pain, with “… *el dolor promedio*,” which better reflects, overall, the degree of pain observed in their pet. Some respondents suggested including in the general activity statement a clarification of what the general activity means in the dog. As this statement does not appear in the original version and would imply a change in the structure of the survey, it was decided not to make any changes. Other respondents suggested changing the item “*el peor dolor*” to “*el dolor más intenso*” or “*el dolor más fuerte*.” Although they mean the same, given that the English translation of “*el peor dolor*” matches the original version (“*pain at its worst*”), it was decided to maintain this latter term.

To ensure these latter changes were understandable and clear to owners, the cognitive debriefing was repeated with five further owners. None of the respondents had objections or suggestions regarding the terms “*en el límite*” or “*promedio*,” and it was decided to leave them in the final version.

A table showing all the items or elements where there was any discrepancy during the translation and cognitive debriefing processes is shown in Appendix B.

## 4. Discussion

The translation and linguistic validation of pain assessment scales into different languages is a fundamental step to achieve their standardized and reliable use in clinical and research settings, without excluding any owner or veterinarian. This allows the collection and comparison of data and studies between different countries. The CBPI has been translated into different languages (1, 11–14) but not to Spanish, which is why a linguistically validated Spanish version of the CBPI has been developed in this work.

To achieve a linguistically validated version of the CBPI, the recommendations and guidelines of WHO and ISPOR have been followed (10, 15, 16). Two native Spanish-speaking linguists were selected for the direct translations, one with technical expertise and familiarity with health sciences, while the other was a person with no relation to the health or veterinary world. Although the guidelines recommend that direct translations be carried out by healthcare personnel, the research team decided to include this second non-technical linguist as it was more reflective of the typical owner in clinical practice. The CBPI is a questionnaire aimed at animal owners who are not necessarily going to have technical expertise. It cannot be ruled out that this decision could have interfered with the translation process. However, given that the unified translation or reconciliation was carried out by veterinarians, comparing the two independent direct translations, and selecting the most appropriate terms or including new ones, it was considered that this fact has not hindered or prevented the translation of the CBPI into Spanish. In some translations of the CBPI (1), two independent linguists native to the target language were selected. In the Italian (13) and French (12) versions the translation was performed by a single person (one of the authors), and subsequently reviewed by three bilingual reviewers. In any case, it was considered that the results obtained were similar leading to linguistically validated versions of the CBPI.

For the cognitive analysis, 50 pet owners (dogs and/or cats) were surveyed and stratified according to age, sex and level of education, to achieve the widest and most diverse population possible and to limit any possible bias. Although the CBPI is intended for owners of dogs with osteoarthritis, the aim was to analyze the comprehension and readability of the Spanish translated version, not the ability of participants to recognize signs or behaviors related to osteoarthritis. Both WHO and ISPOR recommend a minimum of 5 respondents to conduct the cognitive analysis (10, 15, 16), although in this study it was decided to include a larger number to obtain more robust results. The respondents selected included a representative number of people of different ages and gender so that at least two men and two women were included for each age range considered. Besides, all different educational levels considered in Spain were also included.

The process of translation and linguistic validation of CMI must be complemented by psychometric analysis, understood as the construction and evaluation of an instrument developed to assess a non-observable concept such as chronic pain (11), thus demonstrating its reliability, validity, responsiveness, interpretability, etc. In other words, linguistic validation constitutes the first step to guarantee the clinical and research use of scales written in other languages and as a second step, psychometric validation will confirm the usefulness in cultural environments different from the original one. Psychometric validation has been conducted for both the original English version of the CBPI (4, 7, 8, 14), as well as for the French (12), Swedish (11), Italian (13) and Portuguese versions (14).

In conclusion, this study provides a linguistically validated version of the CBPI in Spanish. The availability of a Spanish version will encourage the use of the CBPI by Spanish-speaking veterinarians and researchers for the assessment and management of chronic pain in dogs with osteoarthritis and bone cancer. The next step will be to perform a psychometric validation of the Spanish translation of the CBPI to ensure re-liability and validity.

## Data availability statement

The original contributions presented in the study are included in the article/Supplementary material, further inquiries can be directed to the corresponding author.

## Author contributions

MO, MC, GD, and IG contributed to conception and design of the study. MO wrote the first draft of the manuscript. All authors contributed to the article and approved the submitted version.

## Conflict of interest

The authors declare that the research was conducted in the absence of any commercial or financial relationships that could be construed as a potential conflict of interest.

## Publisher’s note

All claims expressed in this article are solely those of the authors and do not necessarily represent those of their affiliated organizations, or those of the publisher, the editors and the reviewers. Any product that may be evaluated in this article, or claim that may be made by its manufacturer, is not guaranteed or endorsed by the publisher.
