# Supplementary material for: Translation to Spanish and linguistic validation of the Canine Brief Pain Inventory
Source: Front Vet Sci. 2023 Jun 30;10:1203453. doi: 10.3389/fvets.2023.1203453 (PMC10348357; doi:10.3389/fvets.2023.1203453)
Supplement: Supplementary file 1 [file Data_Sheet_1.pdf]

Supplementary Material

Translation to Spanish and linguistic validation of the Canine Brief Pain Inventory (CBPI)

María Olcoz[1*], Miguel Ángel Cabezas[1], Giorgia della Rocca[2] and Ignacio A. Gómez de Segura[1]

*** Correspondence:** Corresponding Author: mariaolcozcordon@gmail.com

1. ***Supplementary Data.*** *Spanish version of the CBPI*

Breve Cuestionario de Dolor Canino

Descripción del dolor

Evalúe el dolor de su perro:

1. Rellene el círculo junto al número que mejor describa **el peor dolor** en los últimos 7 días

   ☐ 0 ☐ 1 ☐ 2 ☐ 3 ☐ 4 ☐ 5 ☐ 6 ☐ 7 ☐ 8 ☐ 9 ☐ 10

   Sin dolor — *Dolor extremo*

2. Rellene el círculo junto al número que mejor describa **el menor dolor** en los últimos 7 días

   ☐ 0 ☐ 1 ☐ 2 ☐ 3 ☐ 4 ☐ 5 ☐ 6 ☐ 7 ☐ 8 ☐ 9 ☐ 10

   Sin dolor — *Dolor extremo*

3. Rellene el círculo junto al número que mejor describa **el dolor promedio** en los últimos 7 días

   ☐ 0 ☐ 1 ☐ 2 ☐ 3 ☐ 4 ☐ 5 ☐ 6 ☐ 7 ☐ 8 ☐ 9 ☐ 10

   Sin dolor — *Dolor extremo*

4. Rellene el círculo junto al número que mejor describa **el dolor que tiene ahora mismo**

   ☐ 0 ☐ 1 ☐ 2 ☐ 3 ☐ 4 ☐ 5 ☐ 6 ☐ 7 ☐ 8 ☐ 9 ☐ 10

   Sin dolor — *Dolor extremo*

Descripción de la función

Rellene el círculo junto al número que mejor describa cómo el **dolor ha interferido en su perro en los últimos 7 días** en:

1. **La actividad general**

   ☐ 0 ☐ 1 ☐ 2 ☐ 3 ☐ 4 ☐ 5 ☐ 6 ☐ 7 ☐ 8 ☐ 9 ☐ 10

   No interfiere — *Interfiere completamente*

2. **El disfrute de la vida**

☐ 0 ☐ 1 ☐ 2 ☐ 3 ☐ 4 ☐ 5 ☐ 6 ☐ 7 ☐ 8 ☐ 9 ☐ 10

No interfiere *Interfiere completamente*

3. **La capacidad para levantarse desde una posición tumbada**

☐ 0 ☐ 1 ☐ 2 ☐ 3 ☐ 4 ☐ 5 ☐ 6 ☐ 7 ☐ 8 ☐ 9 ☐ 10

No interfiere *Interfiere completamente*

4. **La capacidad para caminar**

☐ 0 ☐ 1 ☐ 2 ☐ 3 ☐ 4 ☐ 5 ☐ 6 ☐ 7 ☐ 8 ☐ 9 ☐ 10

No interfiere *Interfiere completamente*

5. **La capacidad para correr**

☐ 0 ☐ 1 ☐ 2 ☐ 3 ☐ 4 ☐ 5 ☐ 6 ☐ 7 ☐ 8 ☐ 9 ☐ 10

No interfiere *Interfiere completamente*

6. **La capacidad para subir escaleras, bordillos, peldaños, etc.**

☐ 0 ☐ 1 ☐ 2 ☐ 3 ☐ 4 ☐ 5 ☐ 6 ☐ 7 ☐ 8 ☐ 9 ☐ 10

No interfiere *Interfiere completamente*

Impresión global

7. **Rellene el círculo junto al número que mejor describa de forma global la calidad de vida de su perro en los últimos 7 días**

☐ Pobre ☐ En el límite ☐ Buena ☐ Muy buena ☐ Excelente

2. ***Supplementary Data.*** *Questionnaire used in the cognitive debriefing process*

Encuesta sobre la legibilidad de la versión española de la escala de valoración de dolor crónico "Cuestionario Breve de Dolor Canino"

Todavía resulta difícil evaluar el grado de dolor crónico en los perros valorando su comportamiento dada nuestra dificultad para interpretar sus emociones. Para reducir la subjetividad de la valoración del dolor se han diseñado cuestionarios sencillos rellenados por propietario.

El **Cuestionario Breve de Dolor Canino** (en adelante CDC; en inglés CBPI) valora la gravedad del dolor de perros y su impacto en su actividad diaria. Consta de diez preguntas, cuatro relacionadas con la gravedad del dolor y seis con su interferencia con actividades diarias. Cada pregunta se mide utilizando una escala numérica de 0 a 10, siendo "*0=sin dolor/el dolor no interfiere*" y "*10=dolor extremo/el dolor interfiere completamente*". También se incluye una pregunta general sobre la calidad de vida del perro en una escala categórica de 5 puntos ("1=pobre", "2=justa", "3=buena", "4=muy buena" y "5=excelente").

Sin embargo, un problema frecuente (incluido el **CDC**) es que han sido desarrollados en inglés y no pueden trasladarse a propietarios de países como España. Para facilitar su uso se ha elaborado una traducción al español, pero debe confirmarse que tiene el mismo significado y las frases son de fácil legibilidad para el propietario medio.

Para verificar dicha legibilidad hemos elaborado tres preguntas sencillas sobre cada pregunta del **CDC** traducida al español, es decir, si la versión traducida es comprensible para el público en general. Es un formulario voluntario y totalmente anónimo. La información aquí obtenida será utilizada para la elaboración de un Proyecto de Investigación y la implantación de una herramienta que consideramos muy valiosa para mejorar la calidad de vida de los perros.

El formulario se divide en dos partes. La primera, con 3 preguntas de información demográfica básica y anónima. La segunda de valoración de cada una de las preguntas del **CDC** para saber si se entiende correctamente o genera algún tipo de ambigüedad o dificultad en su lectura y comprensión y, cuando sea oportuno, se proporcione una alternativa que resulte más adecuada.

Si acepta participar en este proyecto y desea rellenar el formulario, por favor marque la casilla correspondiente a continuación:

☐ Si acepto ☐ No acepto

Muchas gracias por su colaboración.

El formulario comienza en el reverso de esta hoja.

Marque con una X el rango de edad al que pertenece

 20-29 años 30-39 años 40-49 años 50-59 años ≥60 años

Marque con una X el género con el que se identifica

 Hombre Mujer Ninguno de los anteriores

Marque con una X el nivel de estudios

 Primaria Secundaria Bachillerato Estudios universitarios/Formación profesional Estudios de postgrado/Doctorado

---

A continuación, se le van a mostrar uno a uno todos los elementos que componen el **CDC**, y seguidamente se formularán una serie de preguntas sobre el grado de comprensión de cada elemento:

__Descripción del dolor__

Evalúe el dolor de su perro:

1. Rellene el círculo junto al número que mejor describa **el peor dolor** en los últimos 7 días

 0 1 2 3 4 5 6 7 8 9 10

Sin dolor *Dolor extremo*

a. ¿Se entiende correctamente la pregunta, así como las posibles opciones de respuesta?

 Si No

En caso de no comprenderse la pregunta o haber un término que no se entienda indíquelo a continuación:

b. En sus propias palabras, ¿redactaría de alguna otra forma la pregunta de modo que le resultara más sencillo de leer o más comprensible? En ese caso indíquelo a continuación:

2. Rellene el círculo junto al número que mejor describa **el menor dolor** en los últimos 7 días

 0 1 2 3 4 5 6 7 8 9 10

Sin dolor *Dolor extremo*

a. ¿Se entiende correctamente la pregunta, así como las posibles opciones de respuesta?

 Si No

En caso de no comprenderse la pregunta o haber un término que no se entienda indíquelo a continuación:

b. En sus propias palabras, ¿redactaría de alguna otra forma la pregunta de modo que le resultara más sencillo de leer o más comprensible? En ese caso indíquelo a continuación:

3. Rellene el círculo junto al número que mejor describa **el dolor medio** en los últimos 7 días

☐ 0	☐ 1	☐ 2	☐ 3	☐ 4	☐ 5	☐ 6	☐ 7	☐ 8	☐ 9	☐ 10
Sin dolor										*Dolor extremo*

a. ¿Se entiende correctamente la pregunta, así como las posibles opciones de respuesta?

☐ Si ☐ No

En caso de no comprenderse la pregunta o haber un término que no se entienda indíquelo a continuación:

b. En sus propias palabras, ¿redactaría de alguna otra forma la pregunta de modo que le resultara más sencillo de leer o más comprensible? En ese caso indíquelo a continuación:

4. Rellene el círculo junto al número que mejor describa **el dolor que tiene ahora mismo**

☐ 0	☐ 1	☐ 2	☐ 3	☐ 4	☐ 5	☐ 6	☐ 7	☐ 8	☐ 9	☐ 10
Sin dolor										*Dolor extremo*

a. ¿Se entiende correctamente la pregunta, así como las posibles opciones de respuesta?

☐ Si ☐ No

En caso de no comprenderse la pregunta o haber un término que no se entienda indíquelo a continuación:

b. En sus propias palabras, ¿redactaría de alguna otra forma la pregunta de modo que le resultara más sencillo de leer o más comprensible? En ese caso indíquelo a continuación:

Descripción de la función

Rellene el círculo junto al número que mejor describa cómo el **dolor ha interferido en su perro en los últimos 7 días** en:

1. Actividad general

☐ 0	☐ 1	☐ 2	☐ 3	☐ 4	☐ 5	☐ 6	☐ 7	☐ 8	☐ 9	☐ 10
No interfiere										*Interfiere completamente*

a. ¿Se entiende correctamente la pregunta, así como las posibles opciones de respuesta?

☐ Si ☐ No

En caso de no comprenderse la pregunta o haber un término que no se entienda indíquelo a continuación:

b. En sus propias palabras, ¿redactaría de alguna otra forma la pregunta de modo que le resultara más sencillo de leer o más comprensible? En ese caso indíquelo a continuación:

Rellene el círculo junto al número que mejor describa cómo el **dolor ha interferido en su perro en los últimos 7 días** en:

2. Disfrute de la vida

☐ 0 ☐ 1 ☐ 2 ☐ 3 ☐ 4 ☐ 5 ☐ 6 ☐ 7 ☐ 8 ☐ 9 ☐ 10

No interfiere — *Interfiere completamente*

a. ¿Se entiende correctamente la pregunta, así como las posibles opciones de respuesta?

☐ Si ☐ No

En caso de no comprenderse la pregunta o haber un término que no se entienda indíquelo a continuación:

b. En sus propias palabras, ¿redactaría de alguna otra forma la pregunta de modo que le resultara más sencillo de leer o más comprensible? En ese caso indíquelo a continuación:

Rellene el círculo junto al número que mejor describa cómo el **dolor ha interferido en su perro en los últimos 7 días** en:

3. Capacidad para levantarse desde una posición tumbada

☐ 0 ☐ 1 ☐ 2 ☐ 3 ☐ 4 ☐ 5 ☐ 6 ☐ 7 ☐ 8 ☐ 9 ☐ 10

No interfiere — *Interfiere completamente*

a. ¿Se entiende correctamente la pregunta, así como las posibles opciones de respuesta?

☐ Si ☐ No

En caso de no comprenderse la pregunta o haber un término que no se entienda indíquelo a continuación:

b. En sus propias palabras, ¿redactaría de alguna otra forma la pregunta de modo que le resultara más sencillo de leer o más comprensible? En ese caso indíquelo a continuación:

Rellene el círculo junto al número que mejor describa cómo el **dolor ha interferido en su perro en los últimos 7 días** en:

4. Capacidad para caminar

☐ 0 ☐ 1 ☐ 2 ☐ 3 ☐ 4 ☐ 5 ☐ 6 ☐ 7 ☐ 8 ☐ 9 ☐ 10

No interfiere *Interfiere completamente*

a. ¿Se entiende correctamente la pregunta, así como las posibles opciones de respuesta?
 Si No
En caso de no comprenderse la pregunta o haber un término que no se entienda indíquelo a continuación:

b. En sus propias palabras, ¿redactaría de alguna otra forma la pregunta de modo que le resultara más sencillo de leer o más comprensible? En ese caso indíquelo a continuación:

Rellene el círculo junto al número que mejor describa cómo el **dolor ha interferido en su perro en los últimos 7 días** en:

5. Capacidad para correr
 0 1 2 3 4 5 6 7 8 9 10
No interfiere *Interfiere completamente*

a. ¿Se entiende correctamente la pregunta, así como las posibles opciones de respuesta?
 Si No
En caso de no comprenderse la pregunta o haber un término que no se entienda indíquelo a continuación:

b. En sus propias palabras, ¿redactaría de alguna otra forma la pregunta de modo que le resultara más sencillo de leer o más comprensible? En ese caso indíquelo a continuación:

Rellene el círculo junto al número que mejor describa cómo el **dolor ha interferido en su perro en los últimos 7 días** en:

6. Capacidad para subir escaleras, bordillos, peldaños, etc.
 0 1 2 3 4 5 6 7 8 9 10
No interfiere *Interfiere completamente*

a. ¿Se entiende correctamente la pregunta, así como las posibles opciones de respuesta?
 Si No
En caso de no comprenderse la pregunta o haber un término que no se entienda indíquelo a continuación:

b. En sus propias palabras, ¿redactaría de alguna otra forma la pregunta de modo que le resultara más sencillo de leer o más comprensible? En ese caso indíquelo a continuación:

Impresión global

7. Rellene el círculo junto al número que mejor describa de forma global la calidad de vida de su perro en los últimos 7 días

 Pobre Justa Buena Muy buena Excelente

a. ¿Se entiende correctamente la pregunta, así como las posibles opciones de respuesta?

 Si No

En caso de no comprenderse la pregunta o haber un término que no se entienda indíquelo a continuación:

b. En sus propias palabras, ¿redactaría de alguna otra forma la pregunta de modo que le resultara más sencillo de leer o más comprensible? En ese caso indíquelo a continuación:
