# Supplementary material for: Translation to Spanish and linguistic validation of the Canine Brief Pain Inventory
Source: Front Vet Sci. 2023 Jun 30;10:1203453. doi: 10.3389/fvets.2023.1203453 (PMC10348357; doi:10.3389/fvets.2023.1203453)
Supplement: Supplementary file 2 [file Data_Sheet_2.pdf]

Appendix A. Guidelines and recommendations established by the World Health Organization and the International Society for Pharmacoeconomics, and Outcomes Research (ISPOR) have been followed (10, 15, 16)

Table 1. Terminology used in the process of linguistic validation.

- Preparation: initial work carried out before starting the translation process.
- Direct translation: translation from the original or source language into the target language.
- Reconciliation: comparing and merging more than one direct translation into one.
- Reverse or back translation: translation of the new target language version into the source language.
- Review of back-translations: comparison of the back-translated versions of the instrument with the original to highlight and investigate discrepancies between the original and the reconciled version, which is then reviewed in the problem-solving process.
- Harmonisation: comparison of back-translations in various languages with each other and with the original instrument in order to highlight discrepancies between the original and its derivative translations, as well as to achieve a consistent approach to translation problems.
- Cognitive debriefing: testing the instrument on a small group of patients to test alternative wording and verify comprehensibility, interpretation and cultural relevance of the translation.
- Review of the results of the cognitive report and finalisation: comparison of the patients' interpretations of the translation with the original version to highlight and correct discrepancies.
- Proofreading: final revision of the translation to highlight and correct any typographical, grammatical or other errors.
- Final report: written report at the end of the process documenting the development of each translation.

Table 2. Description of the key persons involved in the process.

- Client: the person or group of persons requesting or ordering the translation of an instrument.
- Instrument developer: person or group of persons who developed the original instrument being translated and who may be responsible for the management of the instrument.
- Project manager: the person who coordinates the translation process, working in a CRO (contract research organization) or similar organisation. Provides oversight at each stage of the process.
- Key in-country consultant: the main contact person who manages the process in the target country. This person is (sometimes) responsible for developing the first

direct translation. Must be a native speaker of the target language, fluent in the source language, and must reside in the target country. He/she must have a background in medicine/health/psychology/social sciences and have experience in translating/managing translations of PRO (Patient-Reported Outcomes).

- Direct translators: the persons who carry out the second and subsequent direct translations. They must be professional translators, native speakers of the target language and fluent in the source language. It is preferable that direct translators reside in the target country and have experience in translating PRO measures.
- Independent translator: a translator who can be used to carry out the reconciliation. Must be a native speaker of the target language, fluent in the source language and resident in the target country, preferably with experience in translating PRO measures.
- Back-translators: the people who develop the translations from the target language into the source language. They must be professional translators, native speakers of the language of the source instrument and fluent in the target language. They should have no prior knowledge of the measure and should not see the source or any other version of the language before or during back-translation.
- In-country consultant: person in-country who can be used to conduct cognitive information interviews. Must be a native speaker of the target language, fluent in the source language and resident in the target country, preferably with experience in qualitative interviewing and/or cognitive interviewing techniques.
- Proof-readers: people who check the final translation for typographical, grammatical or other errors. They must be native speakers of the target language.

Appendix B. Items or elements where there was any discrepancy during the translation and cognitive debriefing processes and the final resolution

**Original**	**Independent translations and reconciliation/unified translation**	**Back-translation**	**Review by research team and native English-speaking linguist**	**Cognitive debriefing**	**Final document**
"Rate your dog's pain"	There were differences between the independent translations: "*califica el dolor de tu perro*" and "*valore el dolor de su perro*".<br>Unified translation: "Puntúe el valor de su perro"	"Score your dog's pain	The research team claims that both the direct translation and the back-translation accurately reflect the original version. However, the linguist considered that, although the verbs "rate" and "score" are very similar, a more literal translation and therefore more like the original document would be the verb "*evaluar*"	There were no problems of understanding or suggestions	"*Evalúe el dolor de su perro"*
"Fill in the oval next to the one number…"	There were differences in the independent translations: "*rellene el círculo junto al número…*" and "*Marque con una X el número...".*<br>The authors selected the first option, as the second was far from the original.	"Fill in the circle next to the number…"	The difference between the terms "*círculo*" and "*óvalo*" was not considered to affect the meaning of the translation but given that in Spanish it is much more common to use the term "*círculo*" than "*óvalo*", it was decided to intentionally change this term.	There were no comprehension problems, although there were suggestions for alternative wording of the question: "*Marque el número*", "*Marque el círculo con el número*" and one respondent even suggested that it would be easier to mark boxes with an X. The research team felt that it did not change the meaning of the question or the response options, so there was no need to consult with the linguist and no modifications were made.	"*Rellene el círculo junto al número…"*
"… the pain at its worst in the last 7 days"	There were differences between the independent translations: "… *el dolor más extremo e intenso en los últimos 7 días*" and "… *el dolor en su peor momento en los últimos 7 días*".	"… the worst pain in the last 7 days"	Both the research team and the linguist confirm that both the unified direct translation and the back-translation accurately reflect the original version.	There were no comprehension problems, although there were suggestions for rewording the question: "*Marque el número que mejor describa el dolor más fuerte de los últimos 7 días*", "… *el dolor más intenso*", "… *el dolor más agudo*" and "… *el dolor más intenso que haya*	"*… el peor dolor en los últimos 7 días"*

	Unified translation: “*Rellene el círculo junto al número que mejor describa el peor dolor en los últimos 7 días”*			*experimentado en los últimos 7 días*”. The research team considered that, although there were suggestions as to the wording of this question, it did not pose problems in comprehension and had the same meaning as the translated version, so it was decided not to make any modifications.	
“… the pain at its least in the last 7 days”	There were differences between the independent translations: “… *el dolor menos intenso en los últimos 7 día*s” and “… *el dolor de menor intensidad en los últimos 7 días*”.<br>Unified translation: “*Rellene el círculo junto al número que mejor describa el menor dolor en los últimos 7 días*”	“… the slightest/least pain in the last 7 days”	Both the research team and the linguist confirm that both the unified direct translation and the back-translation accurately reflect the original version.	There were no comprehension problems, although there were suggestions for a different wording of the question: “*Marque el número que mejor describa el dolor más leve*”, el “*dolor más leve*” and “*el dolor menos intenso que haya experimentado en los últimos 7 días*”. The research team considered that, despite suggestions for the wording of this question, it did not pose problems in comprehension and had the same meaning as the translated version, so it was decided not to make any changes.	“… *el menor dolor en los últimos 7 días”*
“… the pain at its average”	There were no differences between the independent translations: “…*el dolor medio en los últimos 7 días*”.<br>Unified translation: “*Rellene el círculo junto al número que mejor describa el dolor medio en los últimos 7 días*”	“… the average pain level in/over the last 7 days”	Both the research team and the linguist confirm that both the unified direct translation and the back-translation accurately reflect the original version.	There was one respondent who did not understand the concept of average pain and another respondent suggested rephrasing the question as “… *el dolor normal*”. The research team felt that this question needed to be translated again. It was decided to change the term “… *dolor medio*”, which is more commonly used in terms of mathematical calculations, to “… *dolor promedio*”, which translates as “average” and is defined as the middle ground or the point at which something is divided in half or almost in half (same definition as the English concept “average”). The cognitive analysis was repeated and none of the participants made any suggestions or	“… *el dolor promedio en los últimos 7 días”*

				modifications of this term, so it was finally decided to keep the word "*promedio*".	
"Ability"	There were discrepancies between the unified translations: "*capacidad*" and "*habilidad*". The research team opted for "*capacidad*" because, although they are synonymous, the English translation was the same as the English version.	"Ability"	No revisions were necessary	There was no problem of understanding or suggestion	*"Capacidad"*
"Ability to rise to standing from lying down"	The two independent translations were very similar: "*Habilidad de levantarse desde una posición tumbada*" and "*Capacidad para levantarse después de estar acostado*".<br>Unified translation: "*Capacidad para levantarse desde una posición tumbada"*	"Ability to stand up from a lying position"	In reconciling the independent translations, there was some hesitation about whether to uses the term "*levantarse*" or "*ponerse de pie*", which in Spanish mean practically the same thing. The research team consulted with the linguist, who commented that it was preferable to use "*levantarse*", which translates as "to stand up" and implies some difficulty or effort, whereas "*ponerse de pie*" (in English it would translate as "get up") does not.	There was no problem of understanding or suggestion	*"Capacidad para levantarse desde una posición tumbada"*
"Overall impression"	There were no differences between the independent translations.<br>Unified translation: "*Impresión general*"	"General impression"	The research team consulted with the linguist, who considered that "general" has a slightly different nuance to what "overall" means, and some details may be lost, and considered that a more similar translation would be "global"	There was no problem of understanding or suggestion	*"Impresión global"*
"Fair"	There were no discrepancies in the independent translations: "*Escasa*". The research team felt that this term did not quite fit the original and decided to replace it with: "*Justa*"	"Fair"	No revisions were necessary	There were no problems of comprehension, but there were suggestions to change this term to: "mediocre" and "*limitada*". These concepts were not used because they were considered to have a more negative connotation than the	*"En el límite"*

				original. The research team finally decided to make a new translation and replace "Justa" with "*En el límite*", as the latter better reflects the concept in English *("fair")*, since "*Justa*" is a concept that is more frequently used in terms of justice, legality or equanimity. The cognitive analysis was repeated and none of the participants made any suggestions or modifications of this term, so it was finally decided to keep "*en el límite*".	
